# Supplementary material for: Learning probability distributions of sensory inputs with Monte Carlo predictive coding
Source: PLoS Comput Biol. 2024 Oct 30;20(10):e1012532. doi: 10.1371/journal.pcbi.1012532 (PMC11524488; doi:10.1371/journal.pcbi.1012532)
Supplement: S1 Appendix — (PDF) [file pcbi.1012532.s001.pdf]

S1 Learning in a linear model with one input neuron and one latent state using MCPC and PC

We compare training the linear model given in figure 1a of the main text using MCPC and PC. We find that MCPC only has equilibrium points at the optimal model parameters while PC does not have an equilibrium for finite weights. This demonstrates the theoretical superiority of MCPC over PC. Additionally, this elucidates why the weights in PC become excessively large which is observed across tasks. Here, we consider a linear model with identity covariance matrices for simplicity. However, the result generally holds for any covariance matrices.

S1.1 Learning with MCPC

Inference. MCPC infers the posterior distribution $p(x_1|y;\theta)$ which for the considered model equals:

$$\begin{aligned} p(x_1|y;\theta) &= \frac{p(y,x;\theta)}{p(y;\theta)} = \frac{\mathcal{N}(y;Wx_1,I)N(x_1;\mu,I)}{\mathcal{N}(y;W\mu,W^2+1)} \\ &= \mathcal{N}\Big(x_1;\frac{W_0y+\mu}{W_0^2+1},\frac{1}{W_0^2+1}\Big). \end{aligned}$$

Expected parameter updates. The expected parameter updates can be found using MCPC's equation for parameter updates given in equations (6) and (7) of the main text. For the weight W_0, the expected parameter update is given by:

$$\begin{aligned} \mathbb{E}\{\Delta W_0\} \propto \mathbb{E}\{\mathbb{E}_{p(x_1|y;\theta)}\{e_0x_1\}\} &= \mathbb{E}\Big\{\int(y-W_0x_1)x_1p(x_1|y;\theta)dx_1\Big\} \\ &= \mathbb{E}\Big\{\int(y-W_0x_1)x_1\mathcal{N}\Big(x_1;\frac{W_0y+\mu}{W_0^2+1},\frac{1}{W_0^2+1}\Big)dx_1\Big\} \\ &= \frac{1}{(w_0^2+1)^2}\Big(-W_0(Var\{y\}+\mathbb{E}\{y\}^2)+\mu(W_0^2-1)\mathbb{E}\{y\}+W_0^3+W_0\mu^2+W_0\Big). \end{aligned}$$

For the prior mean μ, the expected parameter update is given by:

$$\begin{aligned} \mathbb{E}\{\Delta\mu\} \propto \mathbb{E}\{\mathbb{E}_{p(x_1|y;\theta)}\{\epsilon_1\}\} &= \mathbb{E}\Big\{\int(x_1-\mu)p(x_1|y;\theta)dx_1\Big\} \\ &= \mathbb{E}\Big\{\int(x_1-\mu)\mathcal{N}\Big(x_1;\frac{W_0y+\mu}{W_0^2+1},\frac{1}{W_0^2+1}\Big)dx_1\Big\} \\ &= -\frac{W_0}{W_0^2+1}\Big(\mathbb{E}\{y\}-W_0\mu\Big). \end{aligned}$$

In these expressions, $\mathbb{E}\{y\}$ equals the mean of the data and $Var\{y\}$ equals the variance of the data.

Nullclines. The nullclines of the model can be found by equating the expected parameter updates to zero. Therefore, the nullclines for $\mathbb{E}\{\Delta W_0\} = 0$ are given by the expression:

$$\mu = \frac{-(W_0^2 - 1)\mathbb{E}\{y\} \pm \sqrt{(W_0^2-1)^2\mathbb{E}\{y\}^2 + 4W_0^2(Var\{y\} + \mathbb{E}\{y\}^2 - W_0^2 - 1)}}{2W_0}.$$

Similarly, the nullcline for $\mathbb{E}\{\Delta\mu\} = 0$ is given by the expression:

$$\mu = \frac{\mathbb{E}\{y\}}{W_0}.$$

Equilibrium points. Equilibrium points lie at the intersection between the nullclines $E\{\Delta W\} = 0$ and the nullcline $E\{\Delta\mu\} = 0$. By equating the nullclines, you obtain the condition on equilibrium points:

$$W_0^2 = Var\{y\} + 1.$$

The parameter values of the equilibrium points are therefore $\{W_0 = \pm\sqrt{Var\{y\} - 1}, \mu = \pm\frac{\mathbb{E}\{y\}}{\sqrt{Var\{y\}-1}}\}$. These values are optimal because a model with these parameter values has a marginal likelihood $p(y;\theta)$ equal to $\mathcal{N}(y; \mathbb{E}\{y\}, Var\{y\})$.

S1.2 Learning with PC

Inference. For the linear model, PC inference using the dynamics given in equation (14) of the main text converges to the latent state x_1^* for a given input y.

$$\frac{\partial x_1^*(t)}{\partial t} = 0 = -\epsilon_1^* + W_0\epsilon_0^* = -(x_1^* - \mu) + W_0(x_0^* - W_0 x_1^*)$$

$$x_1^* = \frac{\mu + W_0 y}{1 + W_0^2}$$

Expected parameter updates. The expected parameter updates can be found using PC's equation for parameter updates given in equation (16) of the main text. For the weight W_0, the expected parameter update is given by:

$$\begin{aligned}
\mathbb{E}\{\Delta W_0\} &\propto \mathbb{E}\{e_0^* x_1^*\} = E\{(y - W_0 x_1^*)x_1^*\} \\
&= \mathbb{E}\left\{\left(y - W_0\frac{\mu + W_0 y}{1 + W_0^2}\right)\frac{\mu + W_0 y}{1 + W_0^2}\right\} \\
&= \frac{1}{(1 + W_0^2)^2}\Big(W_0\mathbb{E}\{y^2\} + (\mu - W_0^2\mu)\mathbb{E}\{y\} - W_0\mu^2\Big) \\
&= \frac{1}{(1 + W_0^2)^2}\Big(W_0(Var\{y\} + \mathbb{E}\{y\}^2) + \mu(1 - W_0^2)\mathbb{E}\{y\} - W_0\mu^2\Big).
\end{aligned}$$

For the prior mean μ, the expected parameter update is given by:

$$\begin{aligned}\mathbb{E}\{\Delta\mu\} \propto \mathbb{E}\{\epsilon_1^*\} = \mathbb{E}\{x_1^* - \mu\} &= \mathbb{E}\{\frac{\mu + W_0 y}{1 + W_0^2} - \mu\} \\ &= \frac{\mu + W_0}{1 + W_0^2}\mathbb{E}\{y\} - \mu.\end{aligned}$$

Nullclines. The nullclines for $\mathbb{E}\{\Delta W_0\} = 0$ are given, therefore, by the expression:

$$\mu = \frac{-(W_0^2 - 1)\mathbb{E}\{y\} \pm \sqrt{(W_0^2 - 1)^2\mathbb{E}\{y\}^2 + 4W_0^2(Var\{y\} + \mathbb{E}\{y\}^2)}}{2W_0}.$$

Similarly, the nullcline for $\mathbb{E}\{\Delta\mu\} = 0$ is given by the expression:

$$\mu = \frac{\mathbb{E}\{y\}}{W_0}.$$

Equilibrium points. We can attempt to find equilibrium points by finding the intersection between the nullclines for $\mathbb{E}\{\Delta W_0\} = 0$ and the nullcline for$\mathbb{E}\{\Delta\mu\} = 0$ as follows:

$$\begin{aligned}&\frac{\mathbb{E}\{y\}}{W_0} = \frac{-(1 - W_0^2)\mathbb{E}\{y\} \pm \sqrt{(1 - W_0^2)^2\mathbb{E}\{y\}^2 + 4W_0^2(Var\{y\} + \mathbb{E}\{y\}^2)}}{-2W_0} \\ &\mathbb{E}\{y\}\Big(-1 - W_0^2\Big) = \pm\sqrt{\mathbb{E}\{y\}^2(1 - W_0^2)^2 + 4W_0^2(Var\{y\} + \mathbb{E}\{y\}^2)} \\ &\mathbb{E}\{y\}^2\Big(1 + W_0^4 + 2W_0^2\Big) = \mathbb{E}\{y\}^2(1 + W_0^4 - 2W_0^2) + 4W_0^2(Var\{y\} + \mathbb{E}\{y\}^2) \\ &4W_0^2\mathbb{E}\{y\}^2 = 4W_0^2(Var\{y\} + \mathbb{E}\{y\}^2) \\ &Var\{y\} = 0\end{aligned}$$

There are therefore no equilibrium points except when $Var\{y\} = 0$ where all the points on $\mu = \frac{\mathbb{E}\{y\}}{W}$ are fixed points. Consequently, the parameter updates of PC do not converge.
