# Supplementary material for: Learning probability distributions of sensory inputs with Monte Carlo predictive coding
Source: PLoS Comput Biol. 2024 Oct 30;20(10):e1012532. doi: 10.1371/journal.pcbi.1012532 (PMC11524488; doi:10.1371/journal.pcbi.1012532)
Supplement: S2 Appendix — (PDF) [file pcbi.1012532.s002.pdf]

S2 Predictive coding optimizes an infinitely loose bound on the marginal log-likelihood $\ln p(y;\theta)$

Our results suggest that predictive coding fails to learn accurate generative models. This result could be explained by a theoretical shortcoming of predictive coding which is also present in a range of other theories of learning in the brain as discussed below.

Predictive coding learns a generative model by changing its model parameters to maximize the marginal likelihood:

$$p(y;\theta) = \int p(y,x;\theta)\mathrm{d}x$$

Predictive coding uses the variational expectation-maximization algorithm to optimize this marginal which is intractable [1–4]. This algorithm employs the variational distribution $q(x;\phi)$, parametrized by ϕ, to estimate the posterior $p(x|y;\theta)$. It then establishes the free energy $\mathcal{F}$ as an upper bound on the negative log-likelihood:

$$\begin{aligned}\mathcal{F}(\phi,\theta) &= -\mathbb{E}_q\{\ln p(y,x;\theta)\} + \mathbb{E}_q\{\ln q(x;\phi)\} \\ &= -\ln p(y;\theta) + D_{\mathrm{KL}}(q(x;\phi)\|p(x|y;\theta)) \geq -\ln p(y;\theta),\end{aligned} \tag{1}$$

with D_{KL} the KL divergence which is always positive.

The variational expectation-maximization algorithm learns by iterating over two steps. First, it minimizes the free energy $\mathcal{F}$ w.r.t. ϕ for the current parameters θ. In other words, it finds an approximation $q(x;\phi)$ for the posterior. Second, the algorithm minimizes the free energy w.r.t. θ for the parameters ϕ inferred in the first step.

Predictive coding uses the Dirac delta distribution $q(x;\phi) = \delta(x-\phi)$ as variational distribution [3], enabling an implementation of predictive coding using local computation and plasticity. Importantly, the entropy $-\mathbb{E}_q\{\ln q(x;\phi)\}$ for this variational distribution is equal to minus infinity, causing a divergence in the weights of the model [5].

Additionally, the free energy $\mathcal{F}$ minimized by predictive coding is therefore also infinite, making it an infinitely loose bound on the marginal likelihood. This infinitely loose bound raises doubts about the significance of the objective minimized by predictive coding. This theoretical shortcoming may explain the observed poor learning performance of predictive coding in this study despite the application of weight normalization to prevent diverging weights. Moreover, a range of theories for learning in the brain [6–8] are based on a similar energy function as PC's variational free energy, importing the problem of implicitly ignoring an infinite entropy. Future work could therefore explore whether these other learning algorithms exhibit similar suboptimal learning capabilities to those observed in predictive coding.

References

[1] Radford Neal and Geoffrey E. Hinton. *A View of the Em Algorithm that Justifies Incremental, Sparse, and other Variants.* Springer Netherlands, Dordrecht, 1998. ISBN 978-94-011-5014-9.

[2] Dimitris G. Tzikas, Aristidis C. Likas, and Nikolaos P. Galatsanos. The variational approximation for bayesian inference. *IEEE Signal Processing Magazine*, 25(6):131–146, 2008.

[3] Rafal Bogacz. A tutorial on the free-energy framework for modelling perception and learning. *Journal of Mathematical Psychology*, 76(Part B):198–211, 2017.

[4] Karl Friston, James Kilner, and Louise Harrison. A free energy principle for the brain. *Journal of Physiology-Paris*, 100(1-3):70–87, 2006. doi: 10.1016/j.jphysparis.2006.10.001.

[5] Bruno A. Olshausen. Learning linear, sparse, factorial codes. Technical Report AIM-1580, CBCL-138, Massachusetts Institute of Technology, 1996. URL http://hdl.handle.net/1721.1/7184.

[6] Joao Sacramento, Rui P. Costa, Yoshua Bengio, and Walter Senn. Dendritic cortical microcircuits approximate the backpropagation algorithm. In *Advances in Neural Information Processing Systems*, pages 8721–8732, 2018.

[7] James C R Whittington and Rafal Bogacz. An approximation of the error backpropagation algorithm in a predictive coding network with local hebbian synaptic plasticity. *Neural Computation*, 29(5):1229–1262, 2017. doi: 10.1162/NECO_a_00949.

[8] Alexander Meulemans, Nicolas Zucchet, Seijin Kobayashi, Johannes von Oswald, and João Sacramento. The least-control principle for local learning at equilibrium. In *Advances in Neural Information Processing Systems*, volume 35, pages 33603–33617. Curran Associates, Inc., 2022.
