# Supplementary material for: Learning probability distributions of sensory inputs with Monte Carlo predictive coding
Source: PLoS Comput Biol. 2024 Oct 30;20(10):e1012532. doi: 10.1371/journal.pcbi.1012532 (PMC11524488; doi:10.1371/journal.pcbi.1012532)
Supplement: S3 Appendix — (PDF) [file pcbi.1012532.s003.pdf]

S3 Proofs for MCPC capturing the variability of cortical activity

S3.1 Neural variability decreases at stimulus onset

At stimulus onset, the variance of MCPC's steady-state activity decreases. This is because, MCPC's neural activity of the latent state switches from representing the marginal distribution $p(x;\theta)$ to the posterior $p(x|y;\theta)$, which in expectation has a lower variance. The steady state of MCPC's latent state before stimulus onset can be derived similarly to proposition 2 of the main text and equals the marginal distribution $p(x;\theta)$:

$$p^{ss}(x) = \int \frac{e^{-F/\sigma^2}}{Z} dx = \int \frac{e^{\ln p(y=x_0,x;\theta)}}{Z} dx_0 = p(x;\theta) \int \frac{p(x_0|x;\theta)}{Z} dx_0$$
$$= p(x;\theta)$$

Moreover, the law of total variance presented in equation (1) shows that the posterior $p(x|y;\theta)$ has in expectation a lower variance than the marginal distribution $p(x;\theta)$. Therefore, the neural variability of MCPC's latent state is expected to decrease at stimulus onset as experimentally observed in the brain.

$$Var[p(x;\theta)] = E\{Var[p(x|y;\theta)]\} + Var[E\{p(x|y;\theta)\}] \tag{1}$$

S3.2 Natural stimuli specific increase in similarity between spontaneous and evoked neural activity

As the training progresses, the similarity between MCPC's spontaneous activity (encoding the marginal distribution $p(x;\theta)$) and its average evoked activity increases (encoding the posterior distribution $p(x|y;\theta)$). This increase in similarity, however, is specific to the natural stimuli used during training. It does not extend to data outside the training set's distribution, indicating a specialized adaptation to the learned stimuli. This phenomenon can be explained by Bayesian statistics. The marginal distribution $p(x;\theta)$ of a latent variable model with latent state x and input y serves as the model's prior distribution over the latent state, encapsulating prior expectations. Initially, the model parameters are randomly initialized, leading to a discrepancy between the model's average posterior for its inputs and its prior distributions. However, as training advances, the model refines its generative capabilities, aligning the marginal likelihood $p(y;\theta)$ closer to the data distribution $p(y)$. Consequently, the model's prior over the latent state begins to mirror the average posterior distribution more accurately. If the model achieves perfect learning, its prior will match exactly with the expected posterior for the training data. This result follows from Bayesian statistics that specifies that the probability distribution $p(x)$ equals $\int p(x|y)p(y)dy$ (see equation 2). However, the prior of the model is specific to the data distribution. Consequently, when considering posterior distributions for data samples outside the training data distribution, the prior will not equate to the average posterior.

$$p(x) = \int p(x, y) dy = \int p(x|y) p(y) dy \tag{2}$$
