# Supplementary material for: Learning probability distributions of sensory inputs with Monte Carlo predictive coding
Source: PLoS Comput Biol. 2024 Oct 30;20(10):e1012532. doi: 10.1371/journal.pcbi.1012532 (PMC11524488; doi:10.1371/journal.pcbi.1012532)
Supplement: S4 Appendix — (PDF) [file pcbi.1012532.s004.pdf]

S4 Hyperparameter search results

Tables A, B and C section summarises the optimal model parameters for MCPC, PC, and DLGMs found during the hyperparameter search to maximise the FID, the marginal likelihood $-\ln p(y_{eval})$, and the reconstruction MSE.

Table A. Results of the hyperparameter search to maximize the FID for MCPC, PC, and DLGMs.

	MCPC	PC	DLGM
Architecture			
input dimension	748	748	748
number of hidden layers	3	3	3
hidden layers dimension	(128, 128, 20)	(128, 128, 20)	(256,256,20)
activation function	ReLU	ReLU	ReLU
PC Inference			
`optimizer`	Adam	Adam	-
`lr`	0.7	0.1	-
`max_steps`	250	250	-
MCPC Inference			
`optimizer`	SGD	-	-
`lr`	0.1	-	-
mixing steps	50	-	-
sampling steps	100	-	-
Learning			
`optimiser`	Adam	Adam	Adam
`lr`	0.01	0.01	0.001
`decay`	0.0	0.1	0.01
`num_epochs`	50	50	50
`batch_size`	256	128	128

Table B. Results of the hyperparameter search to maximize the marginal likelihood for MCPC, PC, and DLGMs.

	MCPC	PC	DLGM
Architecture			
input dimension	748	748	748
number of hidden layers	3	3	3
hidden layers dimension	(128, 128, 20)	(128, 128, 25)	(128, 128, 10)
activation function	ReLU	Tanh	ReLu
PC Inference			
`optimizer`	Adam	Adam	-
`lr`	0.1	0.3	-
`max_steps`	250	250	-
MCPC Inference			
`optimizer`	SGD	-	-
`lr`	0.03	-	-
mixing steps	50	-	-
sampling steps	100	-	-
Learning			
`optimiser`	Adam	Adam	Adam
`lr`	0.003	0.001	0.001
`decay`	0.1	0.01	0.01
`num_epochs`	50	50	50
`batch_size`	64	256	256

Table C. Results of the hyperparameter search to maximize the reconstruction MSE for MCPC, PC, and DLGMs.

	MCPC	PC	DLGM
Architecture			
input dimension	748	748	748
number of hidden layers	3	3	3
hidden layers dimension	(256, 256, 20)	(256, 256, 30)	(256, 256, 20)
activation function	ReLU	Tanh	ReLu
PC Inference			
`optimizer`	Adam	Adam	-
`lr`	0.7	0.7	-
`max_steps`	250	250	-
MCPC Inference			
`optimizer`	SGD	-	-
`lr`	0.03	-	-
mixing steps	50	-	-
sampling steps	100	-	-
Learning			
`optimiser`	Adam	Adam	Adam
`lr`	0.001	0.003	0.003
`decay`	0.01	0.1	0.1
`num_epochs`	50	50	50
`batch_size`	64	256	128
