# Supplementary figures and images for: Learning probability distributions of sensory inputs with Monte Carlo predictive coding
Source: PLoS Comput Biol. 2024 Oct 30;20(10):e1012532. doi: 10.1371/journal.pcbi.1012532 (PMC11524488; doi:10.1371/journal.pcbi.1012532)

posterior inference

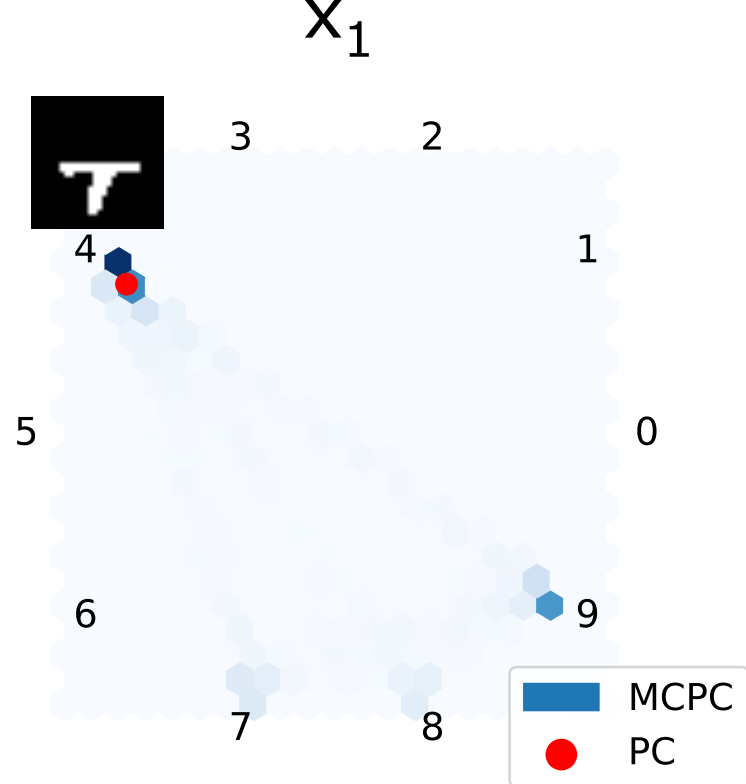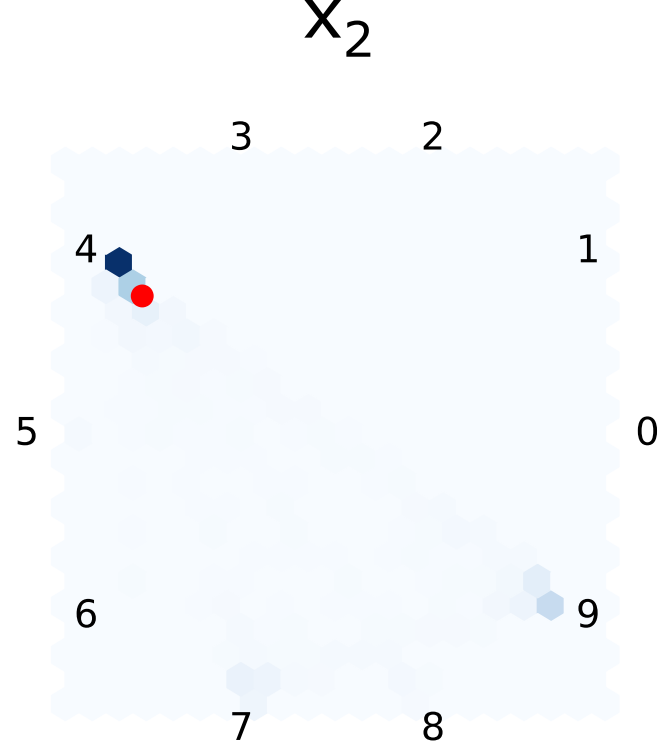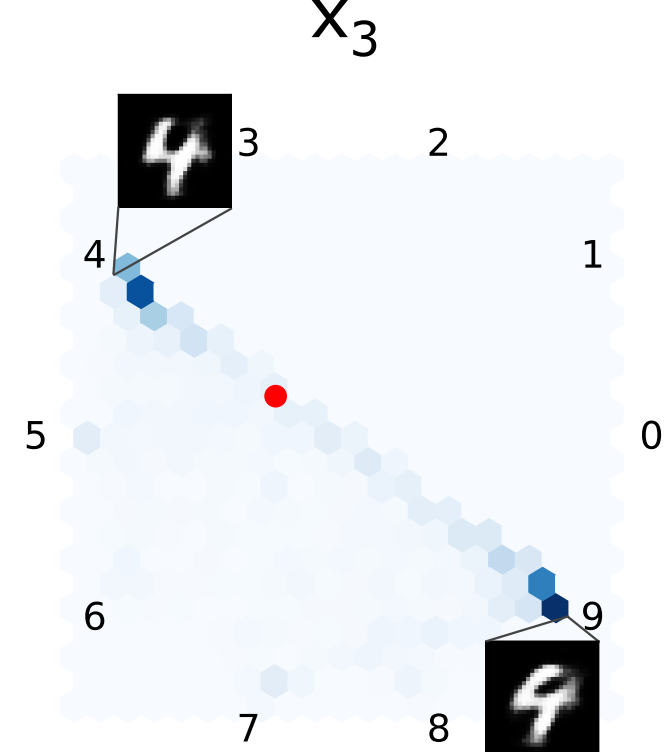

activity similarity

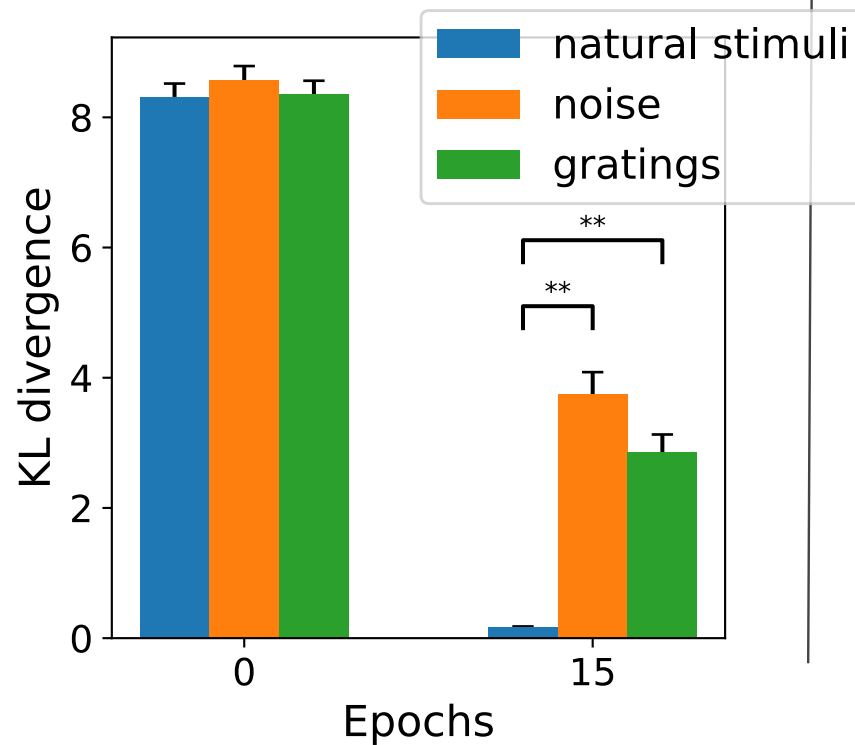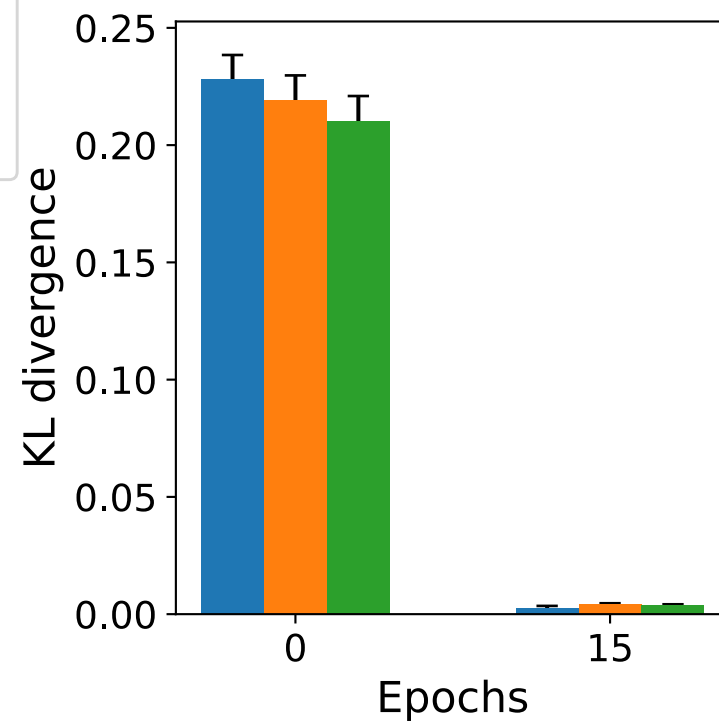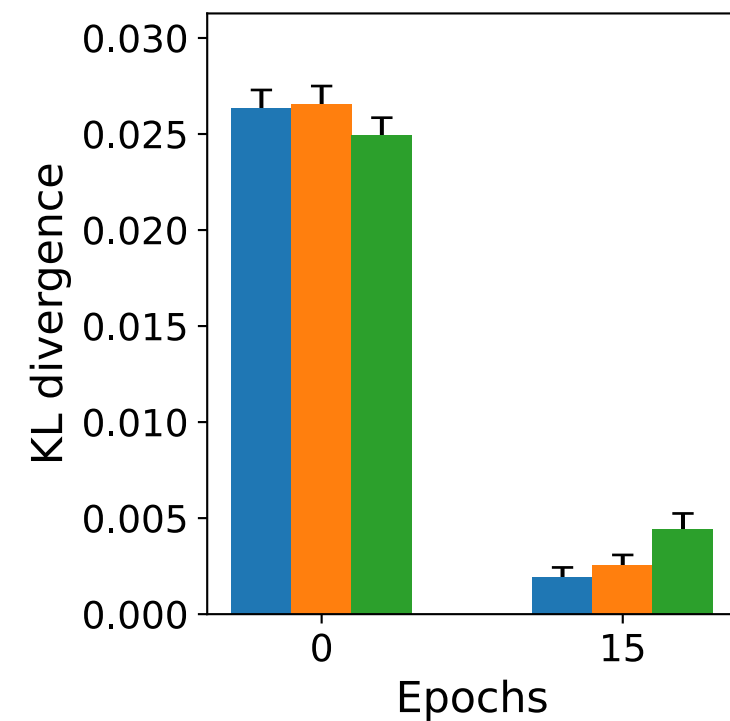

Supplement: S1 Fig — top. We visualize the latent layer activity for a masked input (top left) across all latent layers for PC and MCPC, following the method outlined in Section 4.2.2 of the manuscript. The MCPC model identifies different potential interpretations for a given masked input across its latent layers, whereas the PC model infers only one possible interpretation. Additionally, we visualize the reconstructed images by the MCPC model when the latent layers represent two different possible interpretations. The reconstructed digit when the MCPC model infers a “4” resembles the digit four, while the reconstructed digit when the model infers a “9” resembles the digit nine. bottom. We repeat the analysis to assess the similarity between spontaneous activity and average evoked activity across all latent layers, following the method described in Section 4.3.2. The KL divergence between spontaneous activity and average evoked activity for natural stimuli is lower compared to noise images and image gratings for an MNIST-trained MCPC model across all latent layers. However, this difference is only statistically significant in the first latent layer x1 and not in the higher latent layers. (PDF) [file pcbi.1012532.s005.pdf]

Gaussian task

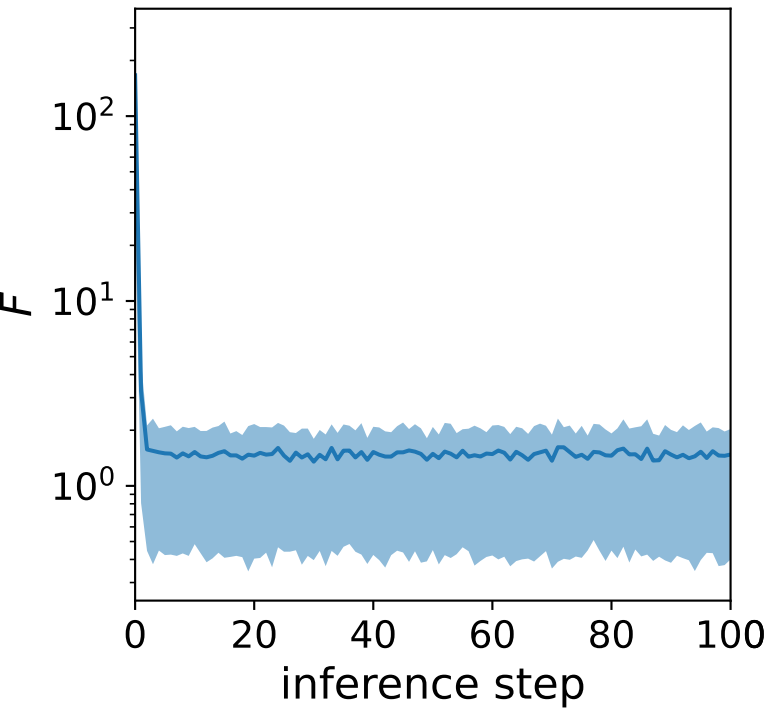

MNIST task

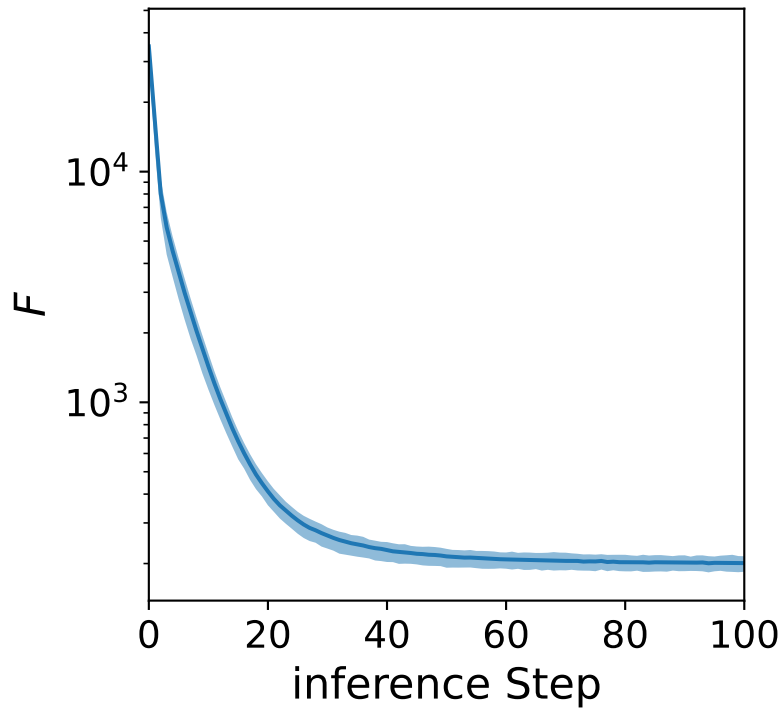

Supplement: S2 Fig — Sampling using Langevin dynamics has been reported to exhibit large mixing times that scale exponentially with the number of dimensions. We employed at least 50 MCPC inference steps along with a PC warmup before sampling for all our experiments. This figure illustrates the negative joint log-likelihood of MCPC models, F=12∑l=0L-1∥xl-Wl·f(xl+1)∥2σ2+12∥xL-μ∥2σ2, during inference averaged for 256 data samples for a model for the Gaussian task {W0 = 2, μ = 0} (left) and of a model trained on MNIST (right). This figure demonstrates that the average sum of prediction errors of the model converges in fewer than 50 inference steps, indicating that the models have likely reached a steady state. This result suggests that MCPC’s convergence time remains manageable even as the size of the latent state increases from one neuron in the Gaussian task to 276 neurons across three layers for the MNIST task. However, larger model might require a convergence time that is beyond practical limits. All the latent variables are randomly initialised before inference following a uniform distribution between -10 and 10. Both models have a learning rate of 0.1 for the activity updates. The shaded region represents the interquartile range. (PDF) [file pcbi.1012532.s006.pdf]

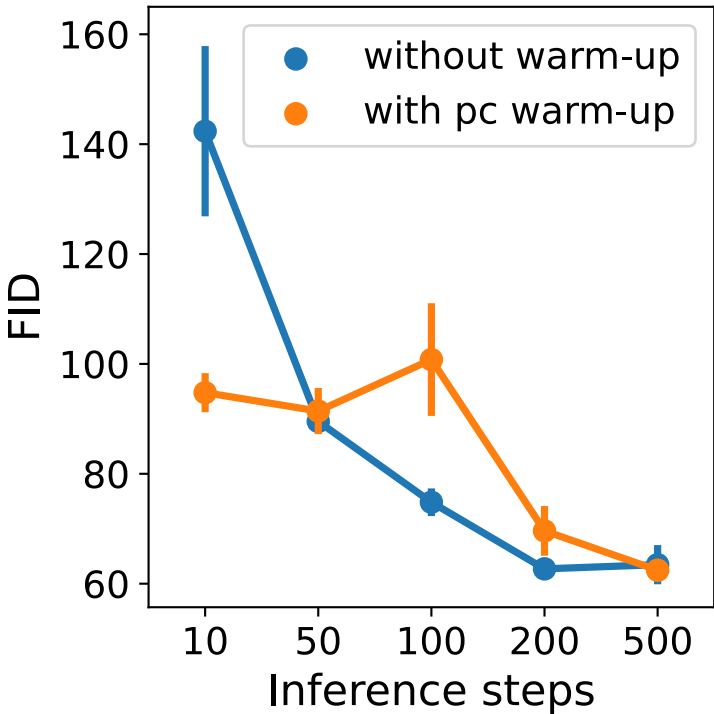

Supplement: S3 Fig — We train an MCPC model on the MNIST dataset with and without warm-up steps. Moreover, we evaluate a range of inference step counts. When the model is trained without warm-up steps, the inference process includes MCPC mixing steps followed by a single MCPC sampling step. However, when the model undergoes training with PC warm-up steps, the initial half of the inference steps consist of PC inference steps, while the remaining inference steps are MCPC mixing steps and one MCPC sampling step. The model parameters for training can be found in S4 Appendix and correspond to the parameters that maximise the FID measure. This figure demonstrates that using PC warm-up inference steps results in improved performance with a limited number of total inference steps, diminished performance with 100 or 200 inference steps, and comparable performance with a large number of inference steps. Ultimately, this result shows that warm-up steps are not always beneficial and should be considered for each learning task separately. The results are shown for three initialisation seeds. The dots show the mean result while the error bars show the standard deviation. (PDF) [file pcbi.1012532.s007.pdf]

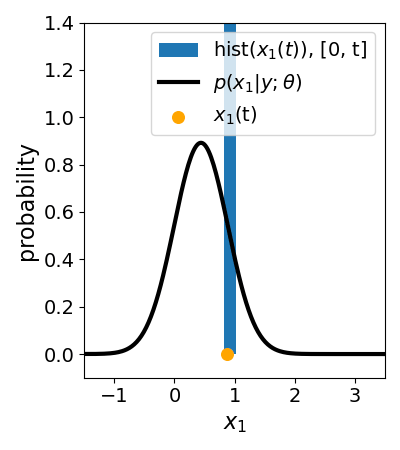

Supplement: S1 Video — Animation of the activity of the latent state in a linear model with one latent state during MCPC inference for a constant input. In this animation, the orange dot shows the time-varying activity of the latent state. The blue histogram summarises the activity of the latent state from the beginning of the animation to the time point in the animation being visualized. Finally, the black curve shows the true posterior distribution that can be analytically calculated from the model parameters and the input to the model. (GIF) [file pcbi.1012532.s008.gif]

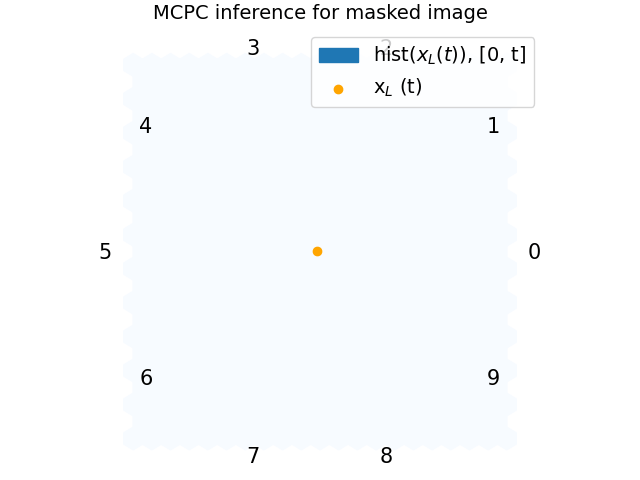

Supplement: S2 Video — Animation of the activity of the latent layer xL in a non-linear model trained on the MNIST dataset during MCPC inference for a half-masked digit input. The orange dot shows the time-varying activity of the latent state xL transformed to coordinates using a linear classifier and a convex combination of 10 evenly spaced points on a unit circle. The linear classifier is trained to decode digit class distributions from the latent state xL. The decoded class distribution can then be transformed to a coordinate using the convex combination. The blue hexagons show the probability density of a two-dimensional histogram of the activity of the latent state from the beginning of the animation to the time point in the animation being visualized. (GIF) [file pcbi.1012532.s009.gif]

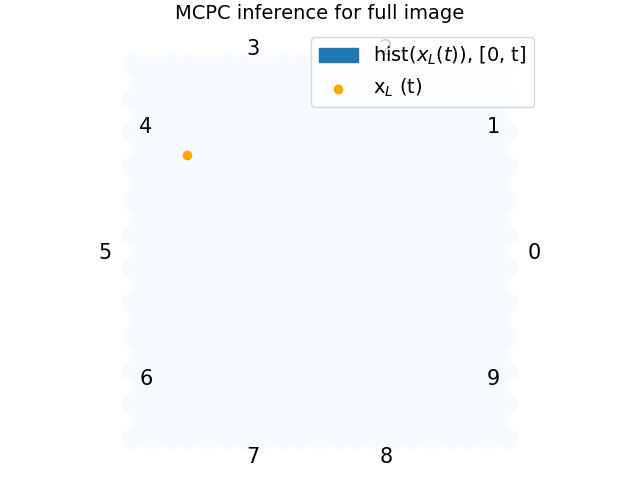

Supplement: S3 Video — Animation of the activity of the latent layer xL in a non-linear model trained on the MNIST dataset during MCPC inference for a full digit input. The orange dot and the blue hexagons have been determined as described in S2 Video. (GIF) [file pcbi.1012532.s010.gif]

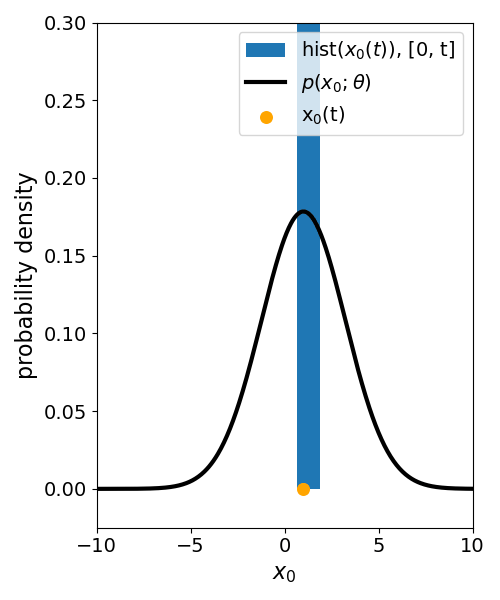

Supplement: S4 Video — Animation of the activity of the input neuron in a linear model with one latent state and one input neuron resulting from MCPC dynamics when the input neuron is unclamped. The orange dot shows the input neuron activity over time. The histogram summarises the activity of the input state from the beginning of the animation to the time point in the animation being visualized. The black curve shows the marginal likelihood that can be analytically calculated from the model parameters. (GIF) [file pcbi.1012532.s011.gif]

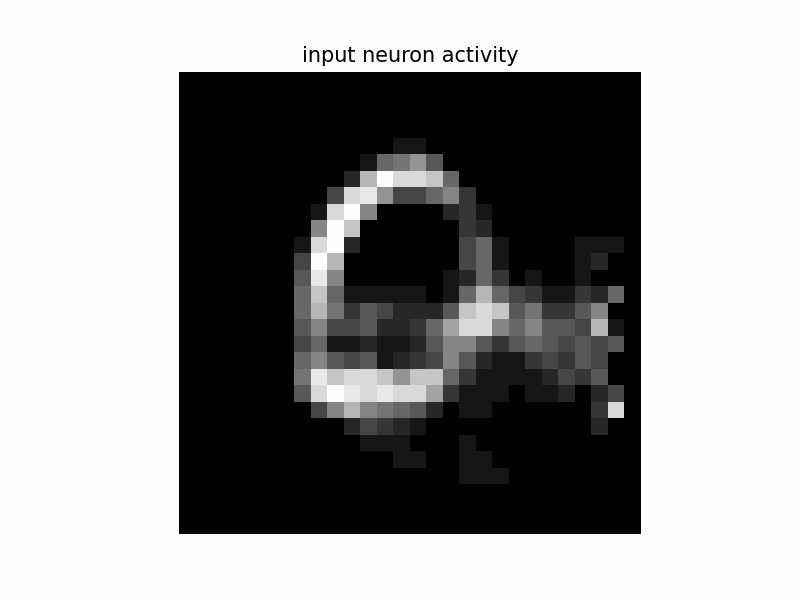

Supplement: S5 Video — Animation of the activity of the input neurons in a non-linear model trained on MNIST resulting from MCPC dynamics when the input neurons are unclamped. (GIF) [file pcbi.1012532.s012.gif]
