# Supplementary material for: Learning probability distributions of sensory inputs with Monte Carlo predictive coding
Source: PLoS Comput Biol. 2024 Oct 30;20(10):e1012532. doi: 10.1371/journal.pcbi.1012532 (PMC11524488; doi:10.1371/journal.pcbi.1012532)
Supplement: Attachment — Submitted filename: MCPC_paper_response.pdf [file pcbi.1012532.s013.pdf]

Response

We thank the Editor and the Reviewers for very useful feedback and suggestions. We addressed all of them, as explained in detail below, and we feel that they significantly improved the manuscript.

Reviewer #1

Major 1. Your understanding and portrayal of predictive coding is colloquial. You are effectively working in a (machine learning) bubble and are discovering well-known aspects of predictive coding. I suspect part of the problem here is that you are not familiar the predictive coding literature beyond machine learning. As such, you cannot appeal to existing formulations, especially in relation to **variational filtering** and its implementation in the brain.

More - I think you need to rewrite your paper starting with a clear definition of what you understand by predictive coding. Predictive coding was introduced in the 1950s for compressing sound files (Elias, 1955) and was first proposed in neuroscience for retinal processing (Srinivasan et al., 1982). Related proposals for hierarchical predictive coding were then discussed in terms of cortical computations and particle filtering (Lee and Mumford, 2003; Mumford, 1992). Hierarchical predictive coding was foregrounded by Rajesh Rao and Dana Ballard (Rao and Ballard, 1999) and subsequently shown to be equivalent to extended Kalman filtering (Friston and Kiebel, 2009; Rao, 1999), which itself is an instance of variational filtering (Friston, 2008b). I mention variational filtering because I think you need to explain how your formulation (e.g., equation 3) differs from the second lemma in (Friston, 2008b). (E.g., equation 12). The answer is that you are considering a limited case in which you have ignored dynamics. In generic predictive coding schemes, one normally works in generalised coordinates of motion. The Kalman filter is an example of just dealing with first-order motion. Your so-called PC is a zero-order approach in which you ignore motion altogether, so that you can focus on static image classification problems. You need to make it clear that your predictive coding is not predictive coding in the general sense. I think you need to find an appropriate acronym; perhaps MLPC for maximum likelihood or machine learning predictive coding?

↪ Thanks for your comment. In response, we have made the following changes:

1. Introduction: We changed the introduction to discuss predictive coding in general and not only predictive coding following Rao and Ballard [1] formulation.

2. Variational filtering in introduction: We made it clear in our introduction how our model relates to variational filtering.

> "··· *MCPC follows the approach of variational filtering [41] by integrating neural sampling into predictive coding, albeit in a simplified variant that disregards the dynamics of stimuli.* ···" — *Page 3, Lines 68-70*

3. Added context to the predictive coding implementation used in our results section: We clarified that PC in our result section refers to the implementation of predictive coding following Rao and Ballard [1]:

> "··· *We compare the properties of our model to predictive coding following the formulation by Rao and Ballard [19] and Bogacz [21] that we refer to with PC.* ···" — *Page 3, Lines 102-104*

> "··· *A comparison between MCPC and other formulations of predictive coding that use techniques such as divisive input modulation [23], free-energy minimisation combined with the Laplace approximation [22], or variational filtering [41] is provided in the discussion.* ···" — *Page 3, Lines 109-112*

4. Added a comparison in discussion: We added a comparison between several implementations of predictive coding in the new discussion section 3.3.

Major 2. Include the variance of layers in the objective function. "encode layer variance in the noise variable to make the dependence between the level of noise in MCPC's dynamics and the variance of its generative layers explicit." This choice is understandable if you thought that it would make it easier for you to reproduce empirical (neurophysiological) responses. However, by failing to incorporate the noise variance (i.e., precision) in your objective function your F is not a "negative joint log likelihood". Your choice is odd given your previous work showing how the noise variance or precision can be itself optimised with respect to F (i.e., variational free energy) (Bogacz, 2017).

More - If you follow through the work on variational filtering you should appreciate that putting the noise variance into the objective function still allows you to link your formulation to stimulus dependent fluctuations in noise levels. This is because when a stimulus arrives the precision weighted prediction errors mean that the curvature of the free energy increases and the excursions around the Langevin flow are attenuated. In other words, the phenomenology you are trying to demonstrate is an emergent property of variational filtering. Put another way, when noise variance increases the curvature of F increases and neurons spread out or diffuse further. This means that if the random fluctuations on motion have unit variance, they will appear to have greater variability when there is no stimulus or there is a loss of precision (by Weber's law). Interestingly, the sample density of neurons – with unit state noise – approximate the posterior density.

I suggest that you test this out using numerical studies. It will be important to do this because the literature on predictive processing emphasises the minimisation of precision weighted prediction errors: e.g., (Ainley et al., 2016; Clark, 2013; FitzGerald et al., 2015; Haarsma et al., 2018; Kanai et al., 2015; Kok et al., 2012; Limanowski, 2022; Shipp, 2016). This is sometimes cast in terms of estimating the Kalman gain in terms of things like the hierarchical Gaussian filter: e.g., (Palmer et al., 2019).

↪ Thank you for your comment. In response, we have made the following changes:

1. **Energy Function Adjustment**: We revised the energy function F to reflect the model's negative log-likelihood, with precision weighted errors based on the layer variance of the hierarchical Gaussian model:

“. . .

$$F = -\ln p(y, x; \theta) = \frac{1}{2} \sum_{l=0}^{L-1} \frac{\|x_l - W_l \cdot f(x_{l+1})\|^2}{\sigma^2} + \frac{1}{2} \frac{\|x_L - \mu\|^2}{\sigma^2} \quad (21)$$

. . . ” — *Page 4, Lines 133-134*

2. **Section 2.6 revisions**: We significantly revised Section 2.6 to clarify that while our model does not learn the layer variance σ^2 (which is constant), it can still learn the variance of the data. An MCPC model learns the data (co)variance within its model weights. We also clarified how the noise variance in MCPC's Langevin dynamics affects learning when it is not set to one. Specifically, the effective variance of the Gaussian layer in MCPC's model changes, but the model remains capable of learning the data distribution, which we illustrate across a range of noise variances. Please see Section 2.6 for the detailed revisions. The following figure has been added to this section to illustrate that the model learns the (co)variance structure of the data.

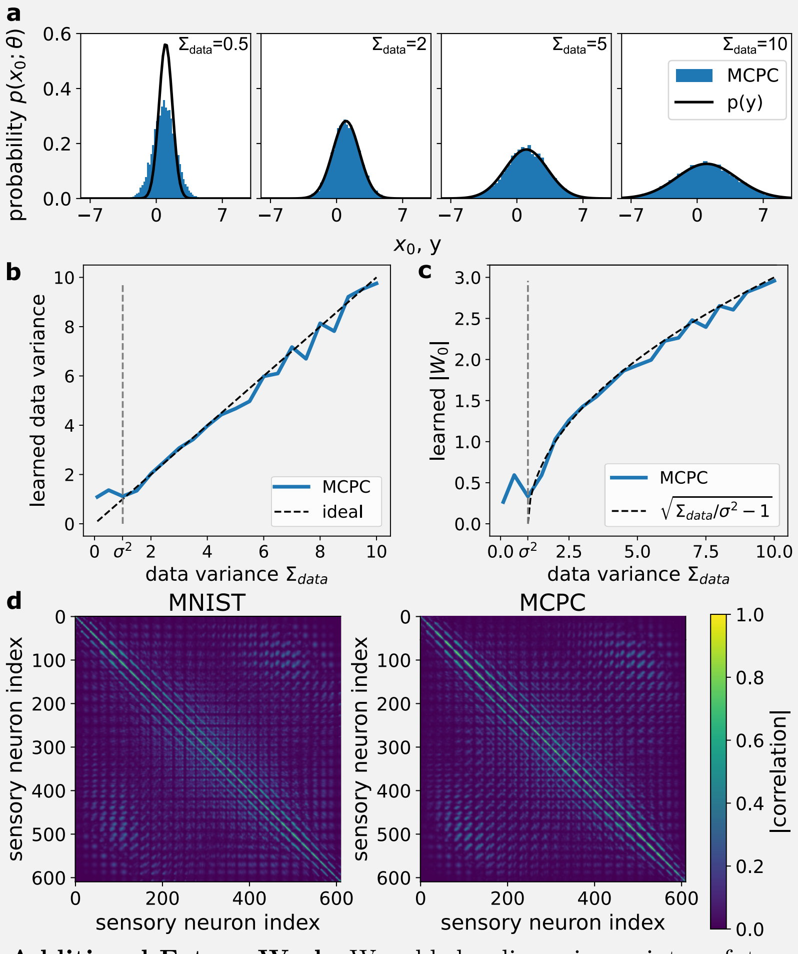

3. **Additional Future Work**: We added a discussion point on future work, suggesting that MCPC could be reformulated to alter the negative log-likelihood

so that the precision matrices of the model's Gaussian layers are also learned. This adjustment is important as learning precision-weighted prediction errors is a central concept in the field of predictive coding, as highlighted by the reviewer. New discussion point:

> "...
>
> #### 4.3.3 Extending MCPC to learn precision weighted prediction errors
>
> *While MCPC effectively learns data variance and covariance over a wide range of data parameters, it encounters learning limitations with narrow data distributions relative to its layer variance. To address this issue, the model could be extended to parameterize and learn the precision matrices of the Gaussian layers within MCPC's generative model, where the precision matrix is the inverse of the covariance matrix. This extension would be particularly significant for the predictive coding field, as existing literature emphasizes the importance of minimizing precision-weighted prediction errors for both computation and neuropathology [25, 26]. Various schemes for predictive coding models have been proposed to learn precision matrices through local computations [21, 46]. These schemes could be applied to MCPC by appropriately modifying the negative log-likelihood function F.*
>
> ..." — *Page 17, Lines 629-641*

Minor 1. Could you nuance your abstract and make it clear that Monte Carlo predictive coding is not a "unification of individual frameworks". Combining predictive coding objective functions with sampling is the basis of particle and variational filtering. I recommend you read about particle filtering and its proposals as a metaphor for stochastic neuronal dynamics in brain hierarchies (Dauwels, 2007; Lee and Mumford, 2003). If you pursue this line of thinking, one can then see that predictive coding without sampling (i.e., viewed as a variational scheme) is simply a description of density dynamics (e.g., variational filtering) under idealised assumptions about sampling. You can find a detailed discussion of this in the companion paper to the variational filtering paper that frames things in terms of dynamic expectation maximisation (Friston et al., 2008). The link between sampling and variational schemes is sometimes expressed as follows: the brain can either be described as performing approximate Bayesian inference exactly (using variational updates) or as performing exact Bayesian inference approximately (using sampling schemes). The former is just a description of the density dynamics you would see under the latter.

↪ Thanks for your comment. We nuanced the abstract to make it clear that MCPC is not the first to unify predictive coding and neural sampling. Moreover, we also added that MCPC is a version of variational filtering for static inputs.

> "... *These frameworks were brought together by variational filtering that introduced neural sampling to predictive coding. Here, we consider a variant of variational filtering for static inputs, to which we refer as Monte Carlo predictive coding (MCPC).* ..."

Minor 2. On page 2, please say "for understanding how the brain infers and learns."

↪ Thanks for your comment. We made the following change to page 2:

> "··· *for understanding how the brain infers and learns.* ··· " — *Page 1, Lines 9-9*

Minor 3. On page 3, please say "by learning efficient generative models, the brain..."

↪ Thanks for your comment. We made the following change to page 3:

> "··· *By learning efficient generative models, the brain establishes the causal relationship between environmental states and sensory inputs [1, 2, 3].* ··· " — *Page 2, Lines 22-24*

Minor 4. "Accuracy" is not the objective. The objective is to maximise the marginal likelihood and ensure an efficient and generalisable model.

↪ Thanks for your comment. In response, we have made the following changes:

1. **Explicit mention of marginal likelihood maximisation**. We explicitly mentioned that our model optimises the marginal likelihood:

> "··· *We demonstrate the precise learning of MCPC by first proving that MCPC is guaranteed to converge to a local optimum of the marginal likelihood* $p(y;\theta)$. ··· " — *Page 8, Lines 276-278*

2. **Rephrased objective**. We rephrase the learning objective of the model. This is shown with the following changes:

> "··· *MCPC learns efficient generative models that generalise from sensory data.* ··· " — *Page 3, Lines 95-95*

3. **MCPC is capable of generalisation**. We conducted additional experiments that show that MCPC learns hierarchies of abstraction, indicating MCPC's ability for generalisation. The hierarchies of abstraction are illustrated by the features learned by an MCPC model on MNIST shown below. This result was added to and is discussed in the results section 2.4.

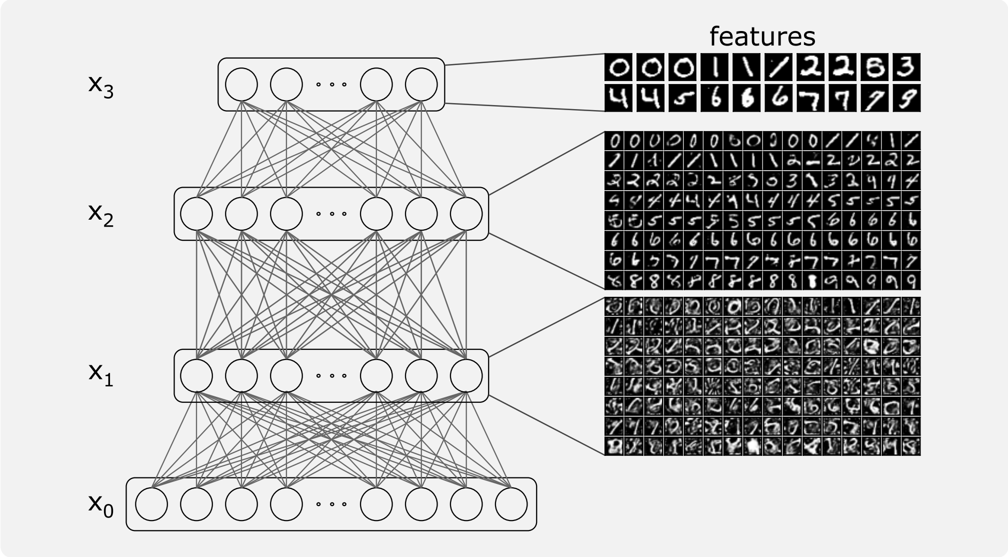

Minor 5. At the bottom page 3, please remove assertions that models implementing the Bayesian brain principle do not adhere to your three constraints. There are numerous examples that contradict this assertion. You can find a review in (Friston, 2008a)

↪ Thanks for your comment. We removed the assertion that models implementing the Bayesian brain principle do not adhere to your three constraints on Page 2, Lines 36-36.

Minor 6. On page 4, please remove any intimation that predictive coding ”only infers the most likely state of the environment from sensory inputs”. It may be that your implementations were restricted in this way. However, generalised predictive coding schemes estimate a full posterior over both states and parameters. (Please see below).

↪ Thanks for your comment. We removed the intimation that predictive coding only infers the most likely state of the environment on Page 2, Lines 48-48.

Minor 7. On page 18, you are correct to say that the expectation maximisation scheme is equivalent to a Dirac delta function over the true posterior: but not over the latent states, rather over model parameters. The notion that sampling ”optimises a tightly bound on the log p(y ; theta) (proposition 3) is not an explanation for the failures of expectation maximisation. The failures of variational expectation maximisation relate to the fact that it ignores uncertainty about the parameters. In other words, in contrast to generic predictive coding schemes, machine learning implementations assume a point mass over the parameters and are therefore unable to evaluate the marginal likelihood that needs to be optimised; i.e., p(y). This is a subtle but fundamental issue which confounds much of machine learning — but is resolved in predictive coding.

↪ Thanks for your comment. The failure of variational expectation maximisation using a Dirac delta function as variational distribution indeed relates to the fact that it ignores uncertainty. Consequently, the marginal likelihood that needs to be optimised can not be evaluated.

We made the following corrections:

1. **Rephrased explanation of failure mode**: We clarified the explanation of the failure mode of predictive coding using Dirac delta functions. We explicitly mentioned that the marginal likelihood can not be evaluated. We also clarified why it can not be evaluated (because entropy term of the free energy is infinite making the free-energy a loose bound on the marginal likelihood):

> *"··· Crucially, the Dirac-delta ignores uncertainty and introduces an infinite entropy to the free-energy, causing the free-energy to become an arbitrarily loose bound on* $\ln p(y;\theta)$ *(refer to Olshausen [57] and S2 Appendix for additional details). As a consequence, the marginal likelihood that needs to be optimised can not be evaluated, which in practise leads to PC's weights diverging. ···" — Page 10, Lines 330-334*

2. **Rephrased mention of proposition 3**: We rephased the mention of proposition 3 because the bound that MCPC optimises depends on the number of inference samples:

> *"··· In contrast, MCPC implements the Monte Carlo expectation-maximisation algorithm that optimises the marginal log likelihood* $\ln p(y;\theta)$ *(Proposition 3). ···" — Page 10, Lines 334-336*

Minor 8. On page 23, you say that "the scalar variance of the noise is not specified in the requirements the fluctuation dissipation theorem." However, this does not mean that "it can be arbitrarily assigned." The noise variance has to match the precision of the data and needs to be estimated in predictive coding. As noted above, your group have done some excellent work along these lines showing how precisions can be estimated in a biologically plausible fashion. I think you need to make this clear and do not leave the reader with the impression that Sigma squared can somehow be assigned arbitrarily.

↪ Thanks for your comment. We rewrote section 2.6 to clarify how the noise variance can be assigned. In the revised section, it should be clear that the variance of the model's Gaussian layers σ^2 and the noise variance σ_n^2 can be assigned a large range of values and the model will still learn the correct generative model. This is because the model adapts its weights as a function of σ^2 and σ_n^2 to learn the correct distribution. However, there are upper limits to the values σ^2 and σ_n^2 can be assigned. Please refer to section 2.6 for changes.

Minor 9. Please remove the assertion on page 27 "Despite these results, PC has encountered challenges in explaining dynamic features of cortical activity". There is a large literature addressing the biological plausibility of predictive coding: e.g. (Shipp, 2016; Walsh et al., 2020). If you are referring to your own work, then you need to change the acronym PC. It may be that you are referring to your static implementation which, by definition, cannot explain "dynamic features of cortical activity." If you wanted to refer to future challenges for your formulation, the usual testbed in neurobiology would be mismatch negativity and oddball paradigms, where one introduces prediction errors by experimental design.

↪ Thanks for your comment. We removed the statement that "PC has encountered challenges in explaining dynamic features of cortical activity" on Page 15, Lines 533-533.

Reviewer #2

Major 1. The authors somewhat ambiguously introduce the concept of PC, which can be misleading. PC is a general idea that can be implemented in several ways. One of these approaches is free energy, which the authors refer to. It employs variational inference (VI) to approximate the posterior distribution. The difference between MCPC and free-energy PC is that the authors utilize the Monte Carlo approach instead of VI. I believe the authors should carefully describe the conceptual differences of their idea from existing approaches (e.g., Rao & Ballard, PC/BC-DIM, and free energy).

↪ Thanks for your comment. To make the concept of predictive coding clearer we changed:

1. Introduction: We changed the introduction to discuss predictive coding in general and not only predictive coding following Rao and Ballard [1] formulation.

2. Added context to predictive coding (PC) in results section: We clarified that PC in our result section refers to the implementation of predictive coding following Rao and Ballard [1].

> "··· *We compare the properties of our model to predictive coding following the formulation by Rao and Ballard [19] and Bogacz [21] that we refer to with PC.* ··· " — *Page 3, Lines 102-104*

> "··· *A comparison between MCPC and other formulations of predictive coding that use techniques such as divisive input modulation [23], free-energy minimisation combined with the Laplace approximation [22], or variational filtering [41] is provided in the discussion.* ··· " — *Page 3, Lines 109-112*

3. Added a model comparison in discussion: We added a comparison between several implementations of predictive coding in the new discussion section 3.3.

Major 2. The authors claim that PC cannot infer posterior probabilities. It would be more correct to state that this is true for the implementation of PC within the free-energy framework, where the variational distribution is represented as a delta function. The authors should note that the free-energy framework is not limited to this type of distribution. Moreover, the original implementation employs Laplacian approximation, which is integrated, for example, in the SPM library. In the meantime, I agree that in free-energy-PC there won't be a true posterior distribution for complex models. Unlike free-energy-PC, MCPC does not restrict the type of distribution but requires infinite-time Langevin dynamics simulation. Hypothetically, free-energy-PC could use more complex variational distributions. I believe, such comparisons would help to convey the idea of authors.

↪ Thanks for your comment. We made the following corrections:

1. We removed the statement in our introduction that predictive coding can not infer posterior distributions on Page 2, Lines 48-48.

2. We added a comparison between several implementations of predictive coding in the new discussion section 3.3. We discuss implementations of predictive coding, other than MCPC, that can infer posteriors during inference.

Major 3. MCMC is famous for its convergence time strictly depending on the dimensionality of the parameter space and the hidden states of the generative model. It would be beneficial to address this issue in the article, showing relevant plots.

↪ Thank you for your comment that emphasises that the convergence time of MCMC sampling methods depends on the dimensionality of the latent variables. This means that a larger model requires increased inference times which might become impractical in large models. To address this concern we explicitly added this limitation to the discussion as follows:

> "… *The convergence speed of MCPC's Langevin dynamics also increases with the number of latent dimensions. In our experiments, the model trained on MNIST (over 200 dimensions) requires significantly more inference steps to reach a steady state than the model trained on Gaussian data (1 dimension), as shown in S2 Fig. Scaling MCPC to large models might therefore be limited by the number of inference steps required to reach steady state which may exceed practical limits.* …" — *Page 18, Lines 655-660*

Moreover, we visualised the convergence of MCPC's inference for the tasks considered in the manuscipt and added it to the supplementary materials S5.

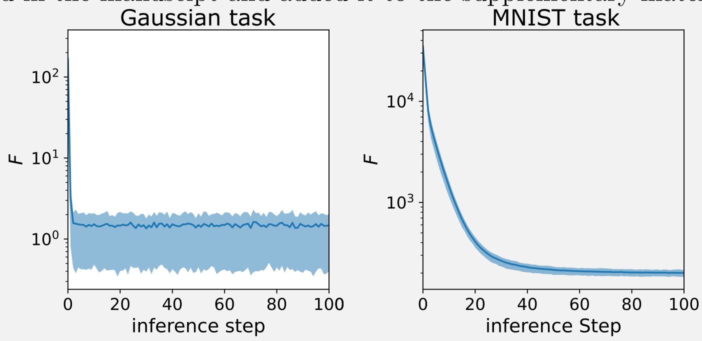

Minor 1. The text doesn't clarify how the model for digit recognition was trained. How do you form the batch? How does the batch participate in the MCPC algorithm? Is it true that each gradient step is averaged over all images, for which Langevin sampling occurs separately and independently?

↪ Thanks for pointing out that the use of batches was poorly explained or defined. To clarify this point we first added an explanation of how batches of data are treated in the methods section 4.1.1 as follows:

> "··· *Moreover, the algorithm learns using mini-batches of data. Inference is performed independently for each element in a mini-batch and parameters are updated using the sum of the parameter updates across the mini-batch.* ··· "
> — *Page 18, Lines 685-687*

We also further clarified how batches of data are used in Algorithms 1 and 2.

Minor 2. The authors mention the potential implementation of a dynamic version (inference of dynamic inputs), but they don't provide details. Since I am skeptical about this possibility, I would like to ask the authors to provide some details regarding this issue.

↪ Thanks for your comment. We reviewed our manuscript to also discuss variational filtering which is a generalisation of MCPC for dynamic inputs. Therefore, we removed the future work section on dynamic inputs. However, we still proposed another method to extend MCPC to dynamic inputs. A recently published study proposed temporal predictive coding (tPC) (`https://journals.plos.org/ploscompbiol/article?id=10.1371/journal.pcbi.1011183`). tPC adds temporal predictions to a predictive coding model that uses Dirac delta as a variational distribution. The resulting model behaves like a Kalman filter that does not track its own posterior variance. We hypothesise that applying Langevin dynamics to a tPC model will bring tPC closer to a Kalman filter by enabling posterior inference.

Variational filtering and dynamic inputs are discussed in the new discussion section 3.3 in the following paragraph:

> "··· *Finally, variational filtering represents another predictive coding implementation that utilizes Langevin dynamics for inference, similar to MCPC. This approach operates within generalized coordinates, which is beneficial for learning dynamic latent variables and temporal structures in data. MCPC can be considered a zero-order version of variational filtering, where sensory input remains static over time. However, in its current form, MCPC cannot learn dynamic inputs. To extend MCPC to accommodate time-varying inputs, a scheme similar to the recently developed temporal predictive coding [45, 87] could be employed, which uses an additional set of weights to predict future latent states from past latent states.* ··· "
> — *Page 16, Lines 562-570*

Minor 3. In line 286, what does "locally optimal" mean?

↪ Thanks for your comment. To clarify what locally optimal means in this context we rephrased "locally optimal" as follows:

> "··· *We demonstrate the precise learning of MCPC by first proving that MCPC is guaranteed to converge to a local optimum of the marginal likelihood* $p(y;\theta)$. ··· "
> — *Page 8, Lines 276-278*

Reviewer #3

Major 1. My biggest worry is that most of the results presented here are, respectfully, not entirely surprising. As the authors noted, the architecture / assumed generative model of MCPC is similar to many previous lines of works that study neural circuits for sampling, especially for one-layer Gaussian generative models. The learning aspect of the model is indeed not common in current literature (especially driven by prediction errors). However, it is not unreasonable to expect EM algorithms would work well in these settings, using Monte Carlo samples. The biological plausibility aspect of the model also mostly inherits predictive coding (local learning rules, error neurons, etc.). Therefore, though I fully believe in the validity of Figure 2-4 presented by the authors, they do not convince me with extra insights provided to neuroscience audiences about the neural circuits, more than an implementation of generative model.

↪ Thank you for your comment. We appreciate the reviewer's feedback and would like to address the concerns about the novelty and insights of our work:

Novelty of Learning Mechanism: While EM algorithms are known and have been applied to predictive coding, predictive coding models with local computations that perform well on generative learning tasks are not present in current literature. Our model shows that integrating prediction error-driven learning and neural sampling addresses this issue.

Insights into Neural Circuits: Our model minimally modifies the neural dynamics and weight updates of Rao and Ballard [1] formulation of predictive coding (additional noise in dynamics only). As a consequence, our model offers the new perspective that the several neural circuits proposed for Rao and Ballard [1], which were tough only to be able to perform MAP inference, can perform probabilistic computations in a hierarchical model using local rules.

Biological Plausibility: Previous attempts at formulating a predictive coding model that can perform posterior inference compromised some of the biological plausibility of predictive coding. For instance, the free-energy principle implementation of predictive coding [2] requires non-local computations to compute the uncertainty of an inference and the variational filtering approach to predictive coding [3] requires synchronous sampling of identical models. Here, we propose a model that does not compromise the biological plausibility aspect of predictive coding even if the model is so far limited to static inputs.

Additional insights: Our work provides several additional insights for neuroscience. First, our model can generate learned sensory samples using local neural activity, which is crucial for memory recall—a fundamental brain function. Unlike most generative models that require an external mechanism for sampling, our model achieves this internally. Second, our experiments introduced in this revision (Section 2.6) demonstrate that learning precision matrices in predictive coding models is not required for generative learning performance. Instead, the model weights naturally learn the covariance structure of the data. Finally, our model is robust to various types and levels of noise. This adaptability makes it well-suited as a brain model, as it does not rely on specific noise types and can handle a wide range of noise levels effectively. In contrast, other models of neural sampling in the brain often rely on noise under strict constraints.

We hope these points clarify the contributions and novelty of our work and address the reviewer's concerns.

Major 2. Related to the previous point, in my opinion, the biggest difference of MCPC compared to other sampling models is its **hierarchical structure**, but I do not see many results or discussion about this aspect. For example, an important goal in

hierarchical predictive coding is to learn more and more abstract representations along its hierarchy through prediction and error (e.g. bars/edges → corners → objects). It would be fascinating to see (i) if MCPC can still learn meaningful hierarchies of abstractions, and (ii) how do sampling dynamics at each level unfold when there are ambiguities? The authors showed in Figure 2d that the last layer's latents seem to be bi-modal and encode possible digit identities – what do the other layers look like? Are there multiple modes at other levels or just the last level? I'm also curious to see in the case of the Figure 2d, what does the reconstruction of the digit look like? Are they complete digits? For your results in Figure 5, which level's activities are these? Are there layer-wise differences?

↪ Thanks for your comment. Investigating the hierarchical structure of the model and its resulting properties is a very interesting research direction.

To this end, we first investigate the features learned by the model trained on MNIST. The figure below shows that an MCPC model trained on MNIST learns hierarchies of abstractions indicating that the model is capable of generalisation.

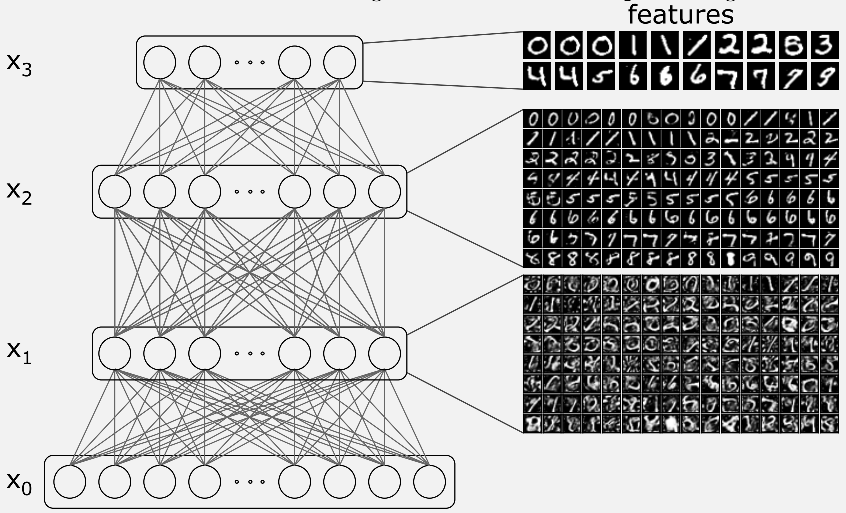

This result was added to the result section 2.4 as an additional figure (figure 5) and the following paragraph discussing it:

"··· *Finally, we demonstrate that MCPC effectively learns hierarchical abstractions from data. Fig 5 illustrates the features learned by all latent neurons in a model trained using MCPC on MNIST. These features are extracted by setting all the neurons' activities in a layer to zero except for one neuron, which is set to a high activity level. The latent activity of that layer is then propagated forward through the model until the input layer is reached. This process is equivalent to finding the activity pattern that minimizes the negative joint log-likelihood conditioned on the manipulated layer of neurons. The features learned by the first latent layer, x_1, consist of 128 low-level features. The features learned by the second latent layer, x_2, include 128 digit representations that vary in orientation, shape, and style. The features learned by the final latent layer, x_3, consist of 20 digits encompassing most classes with minimal within-class variation. This progression indicates that the model learns increasingly abstract features from the input layer to the deepest latent layer. Furthermore, this demonstrates the model's ability to transition from representing pixel-level information in the lower layers to capturing semantic information in the higher layers, thereby showcasing its capacity for generalization.* ···" — *Page 10, Lines 360-375*

An explanation of how the features were obtained was also added in the Methods section 4.2.2:

"··· ***Visualising model features.*** *We visualize the model features of all latent neurons for a model trained on MNIST with MCPC with hyperparameters that optimize the model's FID. The feature represented by a neuron is determined by setting the activity of that neuron to 10 while keeping all other neurons in the same layer at zero activity. This approach is due to the use of ReLU non-linearities in the model, which disregard any activities less than or equal to zero. The chosen activity level of 10 for the analyzed neuron is based on experimental findings showing that activities of 10 or higher produce the same generated pattern. The neuron's feature is obtained by propagating the modified layer activity forward through the model's non-linearities and parameters until it reaches the input layer. For example, after setting the activity in layer x_2 the feature would be calculated as $s(W_0 f(W_1 f(x_2)))$.* ···" — *Page 23, Lines 817-827*

Next, we investigated how sampling dynamics unfold at each level when there are ambiguities and reproduced the results of figure 6b in all layers. This result is shown in the following figure and was added to the supplementary materials S6.

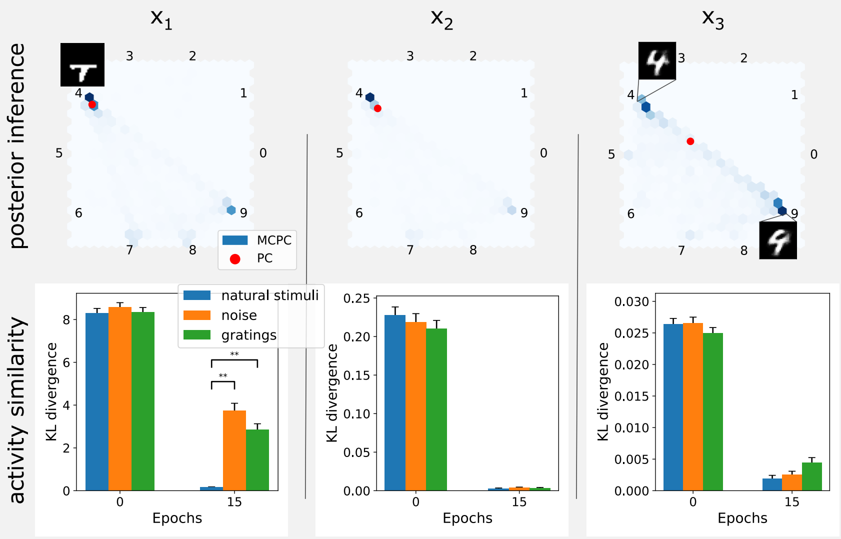

These additional results were added to sections 2.2 and 2.5 as follows

"... *The activity of latent layer x_L is visualised here. However, similar results are observed for all latent layers, as detailed in S1 Fig.* ... " — *Page 7, Lines 220-222*

"... *In our experiment, the similarity in neural activities is measured using the KL divergence for neurons in layer x_1 and natural images are MNIST images (see Methods section 4.2.2 for a detailed explanation of the experiment). The natural stimuli-specific similarity increase is present across the model's latent layers. However, as shown in S1 Fig, the KL divergence is only significantly smaller for natural stimuli in layer x_1.* ... " — *Page 12, Lines 409-414*

Additionally, we clarified that the neural variability of figure 6a is shown for all the latent neurons:

"... *We measure the temporal neural variability by computing the standard deviation of the activity of all the latent states over a sliding window of 1000 timesteps.* ... " — *Page 24, Lines 844-846*

Minor 1 Could the authors explain why the MNIST images were converted to be binary Bernoulli distributions? Would a Gaussian assumption hurt the performance? It seems that there are continuous values (lighter "white" vs. darker "white") in the samples shown in Figure 3, why would this be the case if the input is assumed to binary?

↪ Thanks for your comment. MNIST images have binary pixel values and are therefore not continuous variables but discrete binary random variables. To respect the binary nature of the pixels we opted to use a Bernoulli distribution for layer x_0 instead as it is usually done in generative learning experiments on MNIST. This modeling choice led to improved empirical results, because we incorporated

constraints about the nature of the data, thereby infusing the model with more information. This change does not compromise the biological plausibility of the model because it is equivalent to adding a non-linearity to the model as discussed in section 4.2.2.

Keeping a Gaussian sensory input layer would also be possible. However, it would come with some difficulties. For instance, the variance of the Gaussian input layer clamped to data during training should be very small which could lead to instability during inference. The variance of the input layer should be changed because the variance of individual pixels is much smaller than identity. Some pixels even have no variability.

Thank you for pointing out that the samples shown for our MNIST trained digits are not binary values which you would expect from a Bernoulli distribution. The pixels have continuous values because we show the expected value of the Bernoulli distribution or in other terms the probability of a bernoulli pixel being equal to one. This was done because it is common practice in generative studies on MNIST which makes our result easier to compare to other studies. To make this fact clearer in the manuscript, we first removed the statements that Figure 3 displayed the activity of the input neurons for the model trained on MNIST. We changed the following statement in the figure caption as follows:

> "··· ***b***, *Activity patterns generated by a model trained on MNIST displayed for time points separated by 3,000 timesteps. The samples of the MNIST-trained model display the probability of a sensory neuron being equal to one.* ··· " — *Page 8, Lines 274-274*

and in the result section 2.3:

> "··· *Similarly, Fig 3b illustrates that the unclamped neural activity of a deep non-linear model trained on MNIST produce activity patterns that resemble the digit images used in training.* ··· " — *Page 8, Lines 271-273*

Moreover, we added to the methods section 4.2.2 the following explanation:

> "··· *For the model trained on MNIST, the input Bernoulli sensory layer is discrete and cannot utilize Langevin dynamics. Therefore, we record the neural activity of the model excluding the Bernoulli sensory layer and display the input to the Bernoulli layer predicted by the first latent layers. Mathematically, this is represented as* $s(W_0 f(x_1))$*.* ··· " — *Page 22, Lines 774-779*

Minor 2 In 4.1.1 (and Algorithm 1) there is a "warm-up" step that first performs K steps of gradient descent (without noise) before transitioning to Langevin sampling with noise – is this step necessary? I'm curious if this would actually lead to worse sampling performance (especially in high dimension), if one particular mode of the posterior has a large basin.

↪ Thanks for your observation that having "warm-up" steps without noise might lead to worse sampling performance for high-dimensional models. We added these warm-up steps because preliminary experiments showed a slight improvement in model performance with the warm-up steps on MNIST. The FID obtained on MNIST with PC warm-up is 60.6 ± 2.7, while the FID equals 60.9 ± 0.9 for the same model with the same total number of inference steps where the PC warm-up steps are replaced with MCPC inference steps. These results are reported for the model with the parameters that maximise the FID measure.

To further investigate the potential effects of PC warm-up steps on the inference performance, we trained an MCPC model on the MNIST dataset with and without warm-up steps. Moreover, we evaluate a range of inference step counts. When the model is trained with warm-up steps, half of the inference steps consist of PC inference steps, while the remaining inference steps are MCPC mixing steps and one MCPC sampling step. However, when the model undergoes training without PC warm-up steps, the PC steps are replaced by MCPC mixing inference steps. For each inference step count the model is trained for 3 model parameter initialisations.

The figure below illustrates that using PC warm-up inference steps results in improved performance with a limited number of total inference steps, diminished performance with 100 or 200 inference steps, and comparable performance with a large number of inference steps. Ultimately, this result shows that warm-up steps are not always beneficial and should be considered for each learning task separately.

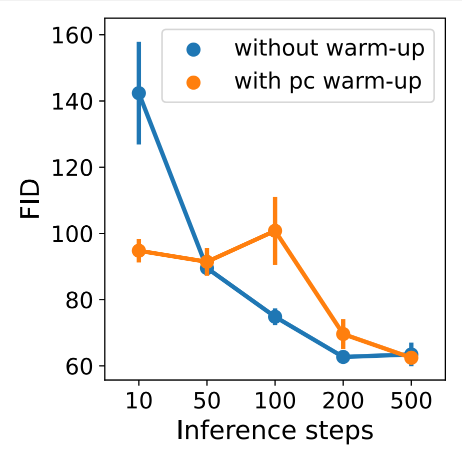

To address this comment in the manuscript, we added the above experiment in Supplementary Information S7 and referred to it in the main body of the paper at:

> "... *This additional MAP inference is, however, not always beneficial as discussed in S3 Fig.* ..." — *Page 18, Lines 684-685*

Minor 3 Equation 2 is missing the top level prior probability x_L

↪ Thank you for pointing out the missing term with the top level prior probability. We added the term in the following equation:

"...

$$F = -\ln p(y, x; \theta) = \frac{1}{2} \sum_{l=0}^{L-1} \frac{\|x_l - W_l \cdot f(x_{l+1})\|^2}{\sigma^2} + \frac{1}{2} \frac{\|x_L - \mu\|^2}{\sigma^2} \tag{22}$$

..." — *Page 4, Lines 133-134*

Minor 4 The authors could unify the symbol for Gaussian pdf (it's N vs. $\mathcal{N}$ at different places..)

↪ Thank you for pointing out that we were inconsistent with our notation for a Gaussian pdf. We unified the notation by always using $\mathcal{N}$.

References

1. Rao RP, Ballard DH. Predictive coding in the visual cortex: a functional interpretation of some extra-classical receptive-field effects. Nature Neuroscience. 1999;2(1):79–87. doi:10.1038/4580.

2. Friston K, Kiebel S. Predictive coding under the free-energy principle. Philosophical Transactions of the Royal Society B: Biological Sciences. 2009;364(1521):1211–1221. doi:10.1098/rstb.2008.0300.

3. Friston KJ. Variational filtering. NeuroImage. 2008;41(3):747–766. doi:10.1016/j.neuroimage.2008.03.017.
